# Suspected Familial Hypertrophic Cardiomyopathy in a 13-Year-Old Male From an Underserved Region of the Dominican Republic: A Case Report

**DOI:** 10.7759/cureus.88861

**Published:** 2025-07-27

**Authors:** Erin M Field, Brian Dickens

**Affiliations:** 1 Department of Family Medicine, Edward Via College of Osteopathic Medicine, Blacksburg, USA

**Keywords:** cardiology, case report, genetics, health disparity, hypertrophic cardiomyopathy, pediatrics, underserved population

## Abstract

In medically underserved communities where longitudinal, coordinated care is limited, patient histories are often complex, with individuals presenting a broad array of concerns and struggling to identify which issues are most urgent. We report the case of a 13-year-old male who presented to our pop-up village clinic with a recent history of episodes of tunnel vision, profuse epistaxis, headache, chest pain, and audible pulse in his ears with both physical and emotional exertion. While at rest in the office, these symptoms were not present. Physical examination showed a thin appearing child presenting with suspected parasitic infection and abnormal cardiovascular findings, including venous distention on the anterior neck and a heart murmur, suggesting a potentially life-threatening condition. Treatment consisted of ivermectin and albendazole for parasitic infection, a multivitamin for nutritional support, and a referral to pediatric cardiology for workup. Here, we describe the challenges of care in an international setting, the importance of gathering a solid history and physical examination, and the disparities associated with hypertrophic cardiomyopathy in underserved communities. This case highlights the importance of access to high-quality healthcare services in underdeveloped and underserved countries to increase patient longevity, improve health equity, and reduce morbidity.

## Introduction

Hypertrophic cardiomyopathy (HCM) is a genetic disease that presents with myocyte disarray, hypertrophy of individual cardiomyocytes, and interstitial and replacement fibrosis. While the historic prevalence of this disease was believed to be one in 500 individuals, the addition of advances in genetic testing, population genetic studies, and diagnostic imaging modalities suggests it may be even more common [1]. According to Arghami et al., with this condition, the most common phenotypic presentation is asymmetric hypertrophy of the interventricular septum, systolic anterior motion of the anterior mitral leaflet, and loss of mitral valve leaflet coaptation [2]. Because of these changes to the heart, pediatric patients may present with left ventricular outflow tract obstruction and mitral valve regurgitation [2]. While left ventricular systolic function is preserved, left ventricular relaxation is impaired [3]. Risks associated with HCM include arrhythmias and sudden cardiac death caused by the presence of a disorganized myocyte pattern, increased wall/lumen ratio of coronaries, and cardiac remodeling [2,4]. Age plays a role in rates of these adverse effects. Those diagnosed before the age of 40 are found to have higher rates of atrial fibrillation, ventricular arrhythmias, and heart failure [5].

While genetics and age play a critical role in cardiovascular health, social determinants of health including education, healthcare access, residential environments, and social context/support play an equally significant role. Based on data from the National Health and Nutrition Examination Survey from 1999 to 2014, in the United States, more favorable social determinant of health scores was associated with more favorable cardiovascular health outcomes. Three of the strongest correlations within this study were poverty level (0.62), household food security (0.57), and access to a routine place for healthcare (0.55) [6]. With unequal health benefits from socioeconomic resources occurring in US adolescents, it is far more likely these are existent in underdeveloped countries. Because of the combination of resource limitations and health disparities in underdeveloped countries, this can make it difficult for patients in these countries to receive adequate diagnoses and proper treatment.

In these underdeveloped countries, where resources and access to healthcare are limited, continuity of care can be difficult. During periods of prolonged absence from medical care, multiple health concerns can accumulate. When a patient finally has access to care, they may report numerous symptoms across various systems, making it difficult to prioritize or distinguish which concerns are most pressing. Having a range of concerns without any recent medical evaluation can make the encounter complicated for both the patient and the healthcare team. In addition, patients in these underserved communities are often incentivized to present with numerous concerns to maximize the medications they can procure for their families as many individuals are unable to afford them otherwise. Due to limitations in resources, families have been known to travel for hours to the pop-up clinics held by our institution, in hopes of acquiring medications and supplies to support their families. This reality adds an additional layer of complexity for the healthcare team, making it more challenging to triage and address the numerous complaints. We present a case of a 13-year-old male with suspected familial hypertrophic obstructive cardiomyopathy in the setting of an underserved community with limited access to healthcare and continuity of care. This case report discusses the pathophysiology, genetics, symptoms, diagnostic procedures, treatment, and disparities associated with hypertrophic cardiomyopathy. The focus of this case report is to describe the challenges of care in an international setting, the importance of gathering a solid history and physical examination, and the disparities associated with hypertrophic cardiomyopathy in underserved communities.

## Case presentation

The patient is a 13-year-old male who presented to a village clinic in the Dominican Republic. This pop-up clinic was held in the classrooms of a local school in the rural Verόn community. The Verόn community consists of a diverse population, including not only individuals from the Dominican Republic, but a large Haitian immigrant population as well. This mixture of individuals created a unique culture consisting of people who spoke different languages (Spanish, Haitian Creole, English), held various cultural values, and came from different socioeconomic backgrounds. Because of the rural setting, transient living situations, and lack of resources, a majority of patients presenting to the clinic had not seen a physician or had any level of continuity of care. With fold-out examination tables, enough medications and supplies to fit into our checked luggage, and the help of local physicians and student translators, community members were provided with access to much-needed healthcare.

**Figure 1:**
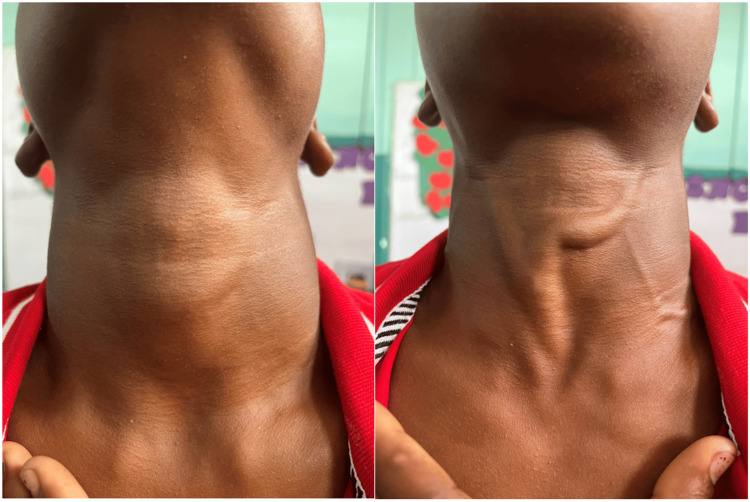
Images of patient’s neck without (left) and with (right) Valsalva maneuver.

At his visit, the 13-year-old male presented with a chief complaint of epistaxis, a lump on the front of his neck, headache for three days, cough, left hip pain, and right elbow pain. His vital signs demonstrated a pulse rate of 61 beats per minute, a respiratory rate of 20 breaths per minute, a blood pressure of 126/52 mmHg, a temperature of 98.4°F, a height of 62 inches, a weight of 100 pounds, and a body mass index of 18.3, which is between the 25% and 50% percentiles based on age. While the patient had no medical record or past medical history, pertinent family history included the sudden death of a half aunt at the age of 23. During review of systems, the patient mentioned that he has a previous history of tunnel vision, profuse epistaxis, headache, chest pain, and pulsatile tinnitus when he either physically or emotionally exerts himself. Examples of scenarios where this occurs include during exercise and when he gets upset with his siblings. While at rest, as the patient was in the office, these symptoms were not present. The patient also explained that he bruises easily and has experienced new onset of abdominal pain, nausea, vomiting, and diarrhea. Recently, the patient began experiencing pain in both his left hip and right elbow while playing baseball, a sport that he plays daily. Because of the lack of continuity of care in these populations, it is typical for patients in these situations to have a lengthy and diverse list of problems they are looking to have addressed. As healthcare professionals, it is our responsibility to differentiate between what is extraneous and what takes priority. During physical examination, the thin appearing male patient was noted to have distended veins on his anterior neck which were more pronounced with the Valsalva maneuver (Figure 1).

The carotid arteries on his neck were also noted to be pulsatile. On auscultation of his heart, a 3/6 systolic murmur was heard at the left upper sternal border, which radiated to his carotid arteries. While squatting, the patient was instructed to stand abruptly while we monitored his heart sounds. The results of a squat-to-stand test showed an increase in intensity of the murmur with the patient standing and a decrease in intensity of the murmur with the patient squatting. In addition, the patient was noted to have palpable, distended temporal vessels as well as pruritic lesions on his hands. Due to the rural setting and limitation of resources, available imaging and further patient workup at the pop-up clinic were limited. Because of these symptoms, physical examination findings, and a family history of young, sudden cardiac death, a mobile point-of-care ultrasound (POCUS) was utilized, a resource the patient would not have had access to otherwise. Ultrasound findings included suspected mild mitral regurgitation, thickened interventricular septum, and left ventricular hypertrophy (Videos 1-3).

**Video 1:** Parasternal short-axis echocardiogram view on mobile point-of-care ultrasound (POCUS).

**Video 2:** Suspected interventricular septum thickening demonstrated by mobile point-of-care ultrasound (POCUS).

**Video 3:** Suspected mitral regurgitation demonstrated by qualitative Doppler view on mobile point-of-care ultrasound (POCUS).

In addition to the 13-year-old male, five of the patient's siblings also presented to the clinic. Of these siblings, two complained of chest pain with exertion. On physical examination, all siblings were found to have either a 2/6 or 3/6 systolic murmur. The second oldest sibling, an 11-year-old female, presented with a 2/6 systolic murmur that was louder upon standing and softer when squatting. Mobile POCUS of the 11-year-old female showed suspected interventricular septum thickening and left ventricular hypertrophy. All six of these siblings share the same mother and father. While the mother did not have a known history of cardiac concerns, the medical history of the father was unknown.

At the end of the appointment, the 13-year-old male was prescribed ivermectin and albendazole for suspected parasitic infection and a multivitamin to support his nutritional needs. The patient, along with his other siblings who also visited the clinic that day, was given a referral to pediatric cardiology for workup. Unfortunately, due to limitations of resources, the patient was unable to be treated with specific medications to address the underlying cardiac findings discovered on presentation at the pop-up clinic. Follow-up with the family was further coordinated by contacts at a major referral center with a pediatric cardiologist available in Punta Cana. Prior to follow-up with pediatric cardiology, we recommended to the patient that he avoid heavy physical activity until a comprehensive evaluation and discussion of risks is completed with an expert.

## Discussion

Genetics and pathophysiology

Hypertrophic cardiomyopathy is inherited in an autosomal dominant pattern, with two-thirds of patients having a family history. While HCM can occur because of variants in a variety of genes, the most common are variants in contractile sarcomere proteins [3]. There have been over 1,500 variants found to cause HCM, with most being unique to each family that presents with this [7]. Due to the phenomena of incomplete penetrance and variable expressivity, phenotypic presentation among affected individuals can differ substantially. This results in patient clinical manifestations ranging from asymptomatic to those with severe heart failure symptoms, angina, arrhythmia, syncope, and sudden cardiac death [7,8].

Echocardiograms are utilized to diagnose hypertrophic cardiomyopathy. According to Basit et al., in children, hypertrophy is defined as a wall thickness that is at least two standard deviations above the mean for age, sex, or body size or a left ventricular wall thickness of 13 mm or more [7]. On histology, HCM presents as enlarged cardiomyocytes that can exhibit unusual shapes, resulting in a disorganized structure of the heart. This disorganization can result in problems with the myocardium and interstitium of the heart, which can lead to the development of left ventricular outflow tract obstruction, mitral regurgitation, diastolic dysfunction, myocardial ischemia, and autonomic dysfunction [7].

Symptoms and physical examination findings

While most children with HCM do not present with symptoms, symptoms in adolescents can include palpitations, chest pain, dyspnea on exertion, syncope, fatigue, and dizziness [9,10]. In addition to these symptoms, some patients with HCM may also present with swelling in their lower extremities or in neck veins [11]. Although our patient’s presentation did not align perfectly with these symptoms, the combination of tunnel vision, headache, chest pain, and pulsatile tinnitus upon exertion is a range of symptoms that clinically make sense for someone experiencing cardiac dysfunction. In cardiac conditions that generate excessive high shear stress in the bloodstream, including HCM, there is the potential to cleave von Willebrand factor (VWF) multimers, leading to an acquired von Willebrand syndrome [12]. This acquired condition could explain epistaxis and easy bruising experienced by our patient. In addition to these findings, our patient presented with extraneous symptoms including pruritus of his hands, hip pain, elbow pain and new-onset abdominal pain, nausea, and diarrhea. Through a detailed history, it was determined these findings were most likely from parasitic infections (findings of pruritus of hands, abdominal pain, nausea, diarrhea) and overactivity while playing baseball (hip and elbow pain). Because of the rural setting and lack of regular healthcare, these findings were unrelated to the suspected cardiac problems but were important to address as well as it was unclear when the patient and his family would have access to medical care again.

According to Basit et al., on physical examination, a harsh mid-systolic ejection murmur can be heard at the left sternal border [7]. This murmur decreases in intensity with squatting and handgrip maneuvers as these maneuvers increase preload or afterload, respectively. This murmur increases in intensity with the Valsalva maneuver or standing, which both cause decreases in preload [7]. By performing a thorough physical examination, we were able to identify the presence of a murmur typically associated with HCM, strengthening our concerns for a cardiac dysfunction and supporting our use of POCUS. In combination with the heart murmur heard, the presence of venous distention on the neck, exaggerated with the Valsalva maneuver, further promotes the likelihood of cardiac strain. Decreased ventricular distensibility can result in marked atrial contraction, causing changes to the jugular venous pulse [13]. When working in these underserved populations with limitations on continuity of care, it is critical to tease through the chief complaints, medical history, and physical examination findings, determining what symptoms and signs require further investigation.

Diagnosis

According to Ommen et al., clinical evaluation of HCM should include collecting a cardiac history and family history, completing a physical examination including the Valsalva, squat-to-stand, passive leg raising, and walking maneuvers, and performing both an electrocardiogram (ECG) and transthoracic echocardiogram [14]. The key information gathered from imaging includes confirming the diagnosis, evaluating for severity, evaluating for other structural and functional abnormalities of the heart, and characterizing the left ventricular outflow tract obstruction [14]. In addition to history, physical examination, and imaging, it is important to rule out other diseases in children that can mimic HCM. These diseases include lysosomal storage diseases, hypertensive heart disease, disorders of fatty acid metabolism, athlete’s heart, cardiac amyloidosis, glycogen storage diseases, mitochondrial cytopathies, and inborn errors of metabolism [15]. Due to a combination of limited access to specialists, advanced diagnostic tools, and continuity of care, diagnosing these diseases can be difficult in resource-limited settings. A key component to ruling out these diseases is newborn screening. Currently, in the Dominican Republic, newborn screening tests are only available upon request from private laboratories [16]. As of April 2024, the Chamber of Deputies in the Dominican Republic passed a bill mandating the implementation of neonatal screening tests in children of the Dominican Republic [17]. While HCM presents at the top of our differential, it remains unconfirmed, and therefore, the presence of the patient’s multiple signs and symptoms, nutritional deficiencies, and physical examination findings could be related to other medical conditions listed within our differential. Narrowing the differential through diagnostic tests is critical and has been found to improve estimation of prognosis, opportunities for genetic counseling, participation in clinical trials, avoidance of ineffective treatments, and refinement of care plans [18]. This further emphasizes the need for access to screening tests, proper diagnostic modalities, and continuity of care in these countries.

Due to limitations of resources, we did not have access to an ECG or transthoracic echocardiogram, key components to obtaining an accurate HCM diagnosis. Through an introductory course on ultrasound, a group of medical students at our institution were trained for over 30 hours in ultrasound through a combination of online modules and the SonoSimulator system (SonoSim, Venice, CA, United States) for guided practice in acquiring ultrasound images. Studies have shown that brief training of medical students in cardiac ultrasound results in significantly more accurate diagnosis of cardiac dysfunction [19,20]. The presence of this training allowed students to utilize POCUS while in the Dominican Republic to improve patient diagnosis and overall care. Utilizing POCUS in this resource-limited population provided a cost-effective strategy to narrow our differential diagnosis, helping to determine the best treatment and follow-up for our patient moving forward. Through development and deployment of point-of-care diagnostics, patients in underserved countries can be provided with accurate, quick, and cost-effective tests and imaging. Although further testing would need to be performed to rule out other diagnoses, the combination of acquiring a solid history, performing a thorough physical examination, and identifying the presence of a thickened interventricular septum with rudimentary POCUS training helps to narrow our differential diagnosis.

Treatment

In those who are asymptomatic, care may consist of observation and monitoring without the need for medical management [5]. For symptom relief, first-line treatment for patients is non-vasodilating beta-blockers and non-dihydropyridine calcium channel blockers as second-line [14]. A core principle of any treatment plan is respecting patient autonomy and ensuring the chosen therapy fits the context of the patient’s life. Through shared decision-making between the healthcare team, patient, and their family, this encourages an improved adherence to the selected treatment, strengthening the likelihood of better health outcomes. In our circumstances, due to limitations of resources available, we were unable to treat the patient with a first- or second-line available option. For this reason, follow-up was scheduled at a major referral center as mentioned previously.

Disparities in diagnosis and treatment of hypertrophic cardiomyopathy

Globally, socioeconomic factors and social disparities have a profound impact on cardiovascular outcomes. According to Borkowski et al., socioeconomic factors that are associated with cardiovascular risk include education, income level, employment status, and environmental conditions [21]. Through combinations of poor diet, psychological distress, and sleep deprivation, both food insecurity and housing insecurity can not only elevate the risk of cardiovascular disease but increase mortality as well. Because of these socioeconomic factors, individuals who live in low- and middle-income countries experience inequalities in disease burden, making up 80% of the global burden of cardiovascular disease [21].

Today, disparities in both the diagnosis and treatment of hypertrophic cardiomyopathy are present between high-income and low-income countries. According to Calderon Martinez et al., those who are in low-income countries face challenges including accessing diagnostic equipment, trained personnel, and affordable medications [22]. If a patient is not diagnosed with HCM until late in its development, the only available treatment option may be invasive procedures, creating a financial burden on these families. In comparison to high-income countries, patients in low-income countries also have disparities in adherence to treatment recommendations. Without early diagnosis or an ability to access the same quality of care as those in high-income countries, patients in low-income countries experience a lower life expectancy [22]. Unfortunately, the patient we are reporting on and his family were put in this position. Due to lack of access, these patients had not received medical care in years and were only able to receive it because of our pop-up clinic in the rural part of the country. While we referred this patient and their family to a pediatric cardiologist, at the time of writing, the family had not followed up or been in further communication with our contacts in the Dominican Republic. Barriers to such follow-up may include economic limitations, geographic distance, poor transportation, and cultural beliefs. In addition, a substantial portion of the Verόn community are undocumented Haitian immigrants, seeking a better life for their families. Because of the fear of deportation for patients and their families, while serving this community, we often experienced resistance from patients to receive further treatment at hospitals within the area.

In underdeveloped countries, the development of dedicated centers focused on management of hypertrophic cardiomyopathy provides an opportunity to step forward in the right direction. According to Maron, through these centers, patients can have access to imaging for diagnosis, genetic/family counselors, and management/treatment options [23]. In India, the Amrita Institute of Medical Sciences and Research in Kochi, Kerala, was established not only with a hypertrophic cardiomyopathy model, but with a well-developed pediatric cardiology section as well. Through the development of centers such as this, the hope is to improve both the longevity and quality of life for patients in underdeveloped areas [23].

## Conclusions

While hypertrophic cardiomyopathy is well addressed in the United States, the presence of global health disparities makes this condition much more difficult to diagnose and treat in other countries. We have presented a case of suspected hypertrophic cardiomyopathy in a 13-year-old male from a rural community in the Dominican Republic. While we were able to treat some of our patient’s initial chief complaints with ivermectin and albendazole for suspected parasites and multivitamins for nutritional support, further workup of our cardiac findings must be completed by a pediatric cardiologist. Because of the combination of a low-resource setting, language barriers, transient patient population, absence of medical records, and lack of continuity of care, this provided additional challenges to care in this already underserved setting. The combination of gathering a detailed history, performing a thorough physical exam, and delivering access to resources otherwise unavailable to these individuals can provide improved care for such populations discussed. Through recognition of symptoms, timely access to diagnostic tools, and prompt treatment, there is an opportunity to improve patient outcomes, both in terms of longevity and quality of life, for underserved community members with medical conditions such as HCM. This case report emphasizes how social determinants of health, including disparities in access to healthcare, significantly impact disease prevention, treatment, and health outcomes. Every child should have the right to access basic healthcare, regardless of where they are born. On a global level, it is our moral responsibility to advocate for these vulnerable populations, providing each child with the opportunity to live a healthy life.
